# SpatialDG: a novel spatial domain identification method for spatially resolved transcriptomics data based on dual-graph neural network

**DOI:** 10.1093/bib/bbag145

**Published:** 2026-04-06

**Authors:** Jiahui Wu, Ayomide Oshinjo, Valerio Izzi

**Affiliations:** Faculty of Biochemistry and Molecular Medicine, University of Oulu, Aapistie 7A, FI-90014 Oulu, Finland; Faculty of Biochemistry and Molecular Medicine, University of Oulu, Aapistie 7A, FI-90014 Oulu, Finland; Faculty of Biochemistry and Molecular Medicine, University of Oulu, Aapistie 7A, FI-90014 Oulu, Finland

**Keywords:** deep learning, contrastive learning, spatial transcriptomics, spatial domain identification

## Abstract

Spatially resolved transcriptomic (ST) technologies offer transformative opportunities to chart gene expression landscapes within intact tissue architecture. Uncovering spatially discrete domains of biological function is essential for deciphering tissue heterogeneity, developmental processes, and disease mechanisms. Yet, the inherent noise, high dimensionality, and spatial sparsity of ST data present substantial challenges to the unsupervised delineation of these domains. We present SpatialDG, a dual-graph self-supervised contrastive learning framework for ST. SpatialDG combines graph neural networks with self-supervised contrastive learning to learn informative and discriminative spot representations by maximizing the agreement between local (node) and global (graph) embeddings and leveraging spatial adjacency to enhance representation learning. Specifically, SpatialDG constructs both a gene expression similarity graph and a spatial adjacency graph, integrating them via a dual-view contrastive architecture that aligns molecular and spatial information, while a zero-inflated negative binomial reconstruction loss accounts for the count-based and sparse nature of gene expression data. SpatialDG achieves significant gains over state-of-the-art algorithms in both healthy and cancer datasets, demonstrating robust generalization across diverse ST landscapes. In conclusion, SpatialDG efficiently unravels biologically meaningful domains from spatial and genetic signals, providing a powerful and generalizable tool to mine tissue architecture in ST datasets.

## Introduction

The spatial architecture of a tissue is a fundamental determinant of its function, governing critical processes in development, homeostasis, and disease pathogenesis. Understanding the precise spatial context of gene expression is therefore crucial for unraveling the complex mechanisms that underpin both normal physiology and pathological states [1]. In this context, spatial transcriptomics (ST) [2] has emerged as a pivotal technology, offering unprecedented insights into the genetic circuits controlling tissue architecture and enabling comprehensive investigation of biological mechanisms, pathogenomic trajectories, and potential therapeutic targets. However, despite the continuous advancements in ST technologies, the accurate identification and definition of spatial domains (tissue regions characterized by similar gene expression patterns and biological function) remains challenging, due to the inherent high dimensionality, dropout, and spatial sparsity of ST data.

Successful clustering of ST data requires both the expression values of informative genes at each spot and the spatial location information of the spot. Yet, methods to detect spatial domains can either involve a spatial component (spatial clustering methods) or not (nonspatial clustering methods and). Nonspatial clustering methods, such as Seurat [3], K-means [4], and Louvain [5], use gene expression data as input and primarily rely on gene expression profiles to group individual spots into distinct domains. These approaches typically involve an initial dimensionality reduction step, employing techniques like principal component analysis [6], t-distributed stochastic neighbor embedding [7], or uniform manifold approximation and projection (UMAP [8]), before applying clustering algorithms to identify putative spatial domains. While capable of identifying clusters based on molecular similarity, a significant limitation of these methods is their inherent disregard for the spatial relationships between neighboring spots. This oversight frequently leads to the identification of spatially discontinuous domains, which often misrepresent the underlying biological organization. Spatial clustering methods explicitly incorporate spatial information during the clustering process to ensure the identified domains are spatially coherent [9]. Spatial clustering methods often combine gene expression, spatial location, and morphology to explain the spatial dependence of gene expression, thereby better matching spatial locations. For example, stLearn [10] uses gene expression data, tissue morphology data, and spatial location information, Giotto [11] uses a hidden Markov random feld [12] model to detect spatial domains based on the spatial dependency between spots, and BayesSpace [13] adopts a Bayesian statistical method that employs the information from spatial neighborhoods to improve clustering analysis.

In recent years, advanced machine learning approaches have demonstrated robust performance across various biomedical tasks, ranging from disease-related compound identification [14] and medical feature reconstruction [15] to functional protein classification [16]. Specific to transcriptomics, integrated architectures have been developed to address inherent data challenges, such as attention-based autoencoders coupled with zero-inflated layers for robust clustering. [17], style transfer models for gene imputation [18], and comprehensive platforms for cell-level investigation [19]. Amidst these advancements, graph neural networks (GNNs) have gained attention for their innate ability to integrate gene expression profiles and spatial information [20]. A variety of heterogeneous graph convolutions have been proposed to exploit spatial graph information, including graph attention network (GAT [21]), sample and aggregate [22], simple graph convolution [23], graph convolutional network (GCN [24]), topology-adaptive graph [25], and unified message-passing model [26]. Many studies have sought to apply these GNNs to partition and identify spatial domains. For instance, SpaGCN [27] uses a GCN-based model to identify spatial domains by integrating gene expression, spatial location, and histology image. STAGATE [28] uses a graph attention auto-encoder framework to identify spatial domains by integrating spatial information and gene expression profiles. CCST [29] generates an embedding for spatial nodes that contains information on both the spatial structure and the gene expressions, then uses a GCN for unsupervised domain clustering. DeepST [30] uses a deep neural network to process morphological images and create a spatially augmented matrix by appending data on gene expression and spatial location, further applying two autoencoders to obtain a latent representation of the augmented data. SEDR [31] identifies the latent representations of genes and embeds spatial data using a variational graph autoencoder framework, while AVGN [32] combines slice images, spatial information, and raw gene expression using a variational graph autoencoder and multi-head attention blocks for spatial domain identification.

In addition to the above methods, where all information is modeled independently across layers, some models have been proposed based on contrastive learning strategies. For example, SpaceFlow [33] uses a contrastive learning strategy and random permutation spatial representation maps to generate negative samples to enhance the learning effect of deep GNNs, and the conST [34] algorithm integrates gene expression, morphology, and spatial information by using a multimodal contrastive learning framework that helps maximizing the information contained in the local and global graph contexts to generate effective latent embeddings. GraphST [35] is a self-supervised graph-based technique for contrastive learning of ST data that encompasses spatial clustering, multi-sample integration, and cell-type deconvolution. The contrastive learning framework in the spatial clustering module learns informative and discriminative representations of spots by minimizing the embedding distance between spatially adjacent spots. Spatial-MGCN [36] utilizes a multi-view GCN with an attention mechanism to identify spatial domains by combining gene expression data and spatial information.

Even the most refined contrastive approaches outlined above, however, still struggle to capture complex multi-scale spatial relationships in biological tissues, particularly when dealing with irregular tissue boundaries, overlapping domains, or transition regions. To overcome these limitations, we develop SpatialDG, an effective spatial domain identification method based on a dual-GNN.

The framework operates through three key stages to integrate molecular and spatial information effectively. First, SpatialDG constructs a dual-graph structure: a feature graph capturing gene expression similarities and a spatial graph encoding physical proximity. These are processed through parallel GCNs with an attention mechanism to adaptively fuse the representations. Second, to handle the sparse and over-dispersed nature of ST data, we employ a zero-inflated negative binomial (ZINB [37]) decoder that explicitly reconstructs gene expression while accounting for dropout events. Third, to enforce global consistency, we incorporate a Dual-View Deep Graph Infomax (DGI) module. This component maximizes the mutual information between individual spots and the global tissue representation, ensuring the learned embeddings are robust to local noise.

The main contributions of this work are summarized as follows:

1. Innovative dual-graph framework: We propose a unified framework that synergizes spatial proximity and gene expression data. By explicitly modeling both views, SpatialDG effectively captures both local geometric structures and global semantic dependencies, resolving the limitations of single-view aggregation methods.
2. DGI: We introduce a specialized DGI mechanism to extract discriminative features. By maximizing the mutual information between spatial and feature views, this module effectively filters out modality-specific noise and learns distinct global patterns, offering a significant advantage over standard self-reconstruction objectives.
3. Cross-platform generalizability: We conduct extensive benchmarking across three distinct spatial transcriptomics platforms: 10x Visium, Stereo-seq, and Slide-seqV2 platforms. SpatialDG consistently outperforms state-of-the-art methods in spatial domain identification and trajectory inference. This multi-platform validation confirms that our framework is robust to varying spatial resolutions and tissue types, overcoming the platform-dependency often seen in existing approaches.

## Materials and methods

### Overview of SpatialDG

**Figure 1:**
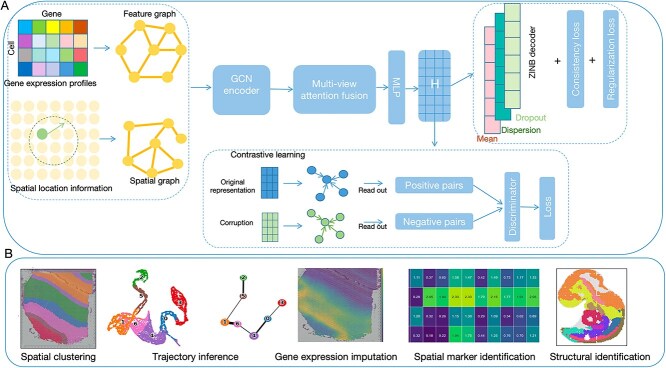
**SpatialDG, a dual-graph contrastive learning framework for spatial data**: (A) Overview of the SpatialDG algorithm; (B) downstream applications of SpatialDG.

In this section, we propose SpatialDG (Spatial Domain identification using dual-GNN), a comprehensive dual-graph contrastive learning framework for spatial domain identification in ST data. The overall architecture is illustrated in Fig. 1A.

Firstly, we construct two complementary graphs to capture distinct aspects of biological information: a feature graph based on gene expression similarity using k-nearest neighbors, and a spatial graph encoding physical proximity relationships through distance-based adjacency. These dual graph setup enables comprehensive modeling of both molecular patterns and spatial organization within tissue samples. Secondly, the graphs are processed through an encoder that employs three specialized GCN components operating synergistically: (i) a feature GCN module that processes the gene expression similarity graph to capture molecular relationships; (ii) a spatial GCN module that leverages spatial proximity information to model neighborhood effects; and (iii) a cross-modal GCN module with shared parameters that operates on both graphs to learn unified representations bridging spatial and molecular domains. To effectively integrate the heterogeneous representations from the three GCN components, we implement an attention-based fusion mechanism that adaptively weights the importance of spatial (H_s), molecular (H_f), and cross-modal (H_c) embeddings, allowing the model to dynamically focus on the most informative features for different tissue regions and spatial domains. Additionally, SpatialDG ships a spatial-aware contrastive learning module that maximizes the mutual information between local node-level and global graph-level representations. Through data augmentation strategies including feature corruption and edge perturbation, the model learns robust embeddings that capture essential biological patterns while being invariant to noise and technical variations commonly present in ST data. The framework then jointly optimizes multiple complementary objectives: (i) ZINB reconstruction loss to handle the zero-inflated and overdispersed nature of spatial transcriptomics data; (ii) contrastive loss for unsupervised representation learning; (iii) consistency loss to ensure coherent cross-modal representations; and (iv) spatial regularization to preserve neighborhood relationships in the learned embedding space. The learned representations (Fig. 1B) serve as a robust foundation for diverse downstream analyses, including spatial domain identification, gene expression imputation, trajectory inference, tumor heterogeneity characterization, and structural analysis of tissue architecture.

### Spatial adjacency graph construction

Spatial adjacency relationships are established based on geometric proximity between locations. The spatial adjacency matrix $\mathbf{A}_{s}^{ij}$ is constructed using pairwise Euclidean distances with a distance-based threshold criterion. Two spots $i$ and $j$ are spatially adjacent if their Euclidean distance $S_{ij}$ falls within radius $r$:


(1) \begin{align*}& \mathbf{A}_{s}^{ij} = \begin{cases} 1, & \text{if } S_{ij} \leq r \\ 0, & \mathrm{otherwise} \end{cases}\end{align*}

The radius parameter is set to $r = 550$ to maintain consistent neighborhood structures across datasets.

#### Feature adjacency graph construction

The feature adjacency graph captures biological relationships between spots based on gene expression profiles using cosine similarity. Given the gene expression matrix $\mathbf{X} \in \mathbb{R}^{n \times d}$ with $n$ spots and $d$ genes, a $k$-nearest neighbors graph $G_{f}=(A_{f}, \mathbf{X})$ is constructed, where $A_{f}^{ij}=1$ if spot $j$ is among the $k$-nearest neighbors of spot $i$, and $A_{f}^{ij}=0$ otherwise. The cosine similarity between expression vectors $\mathbf{x}_{i}$ and $\mathbf{x}_{j}$ is computed as


(2) \begin{align*}& sim(\mathbf{x}_{i}, \mathbf{x}_{j}) = \frac{\mathbf{x}_{i} \cdot \mathbf{x}_{j}}{\|\mathbf{x}_{i}\|_{2} \|\mathbf{x}_{j}\|_{2}},\end{align*}

where $\mathbf{x}_{i} \cdot \mathbf{x}_{j}$ denotes the dot product, and $\|\cdot \|_{2}$ represents the $L_{2}$ norm.

### GCN Encoder

GCNs process graph-structured data by aggregating neighborhood information to capture node dependencies and generate informative embeddings. A multi-view GCN encoder [36] extracts relevant information from both gene expression and spatial structure through convolution operations on feature and spatial graphs. The encoder comprises four components: spatial convolution, feature convolution, co-convolution, and attention mechanism.

#### Spatial convolution

To integrate gene expression with spatial information, convolutional operations are performed on the spatial affinity matrix $\mathbf{A}_{s}$ to aggregate spatial information of neighbors in the Fourier domain. The multilayer spatial convolutional network applies the following hierarchical propagation rule:


(3) \begin{align*}& \mathbf{H}_{s}^{(l+1)} = \mathrm{ReLU}\left(\widetilde{\mathbf{D}}_{s}^{-\frac{1}{2}} \widetilde{\mathbf{A}}_{s} \widetilde{\mathbf{D}}_{s}^{-\frac{1}{2}} \mathbf{H}_{s}^{(l)} \mathbf{W}_{s}^{(l)}\right),\end{align*}

where $\mathbf{W}_{s}^{(l)}$ represents the weight matrix of the $l$th layer in spatial convolution, with initial condition $\mathbf{H}_{s}^{(0)} = \mathbf{X}$ and ReLU activation function. The normalized adjacency matrix is defined as $\widetilde{\mathbf{A}}_{s} = \mathbf{A}_{s} + \mathbf{I}$, where $\widetilde{\mathbf{D}}_{s}$ is the corresponding diagonal degree matrix.

#### Feature convolution

To learn comprehensive gene expression information and impute missing expression values in the feature graph, feature convolution is performed on the feature affinity matrix $\mathbf{A}_{f}$ and gene expression matrix $\mathbf{X}$:


(4) \begin{align*}& \mathbf{H}_{f}^{(l+1)} = \mathrm{ReLU}\left(\widetilde{\mathbf{D}}_{f}^{-\frac{1}{2}} \widetilde{\mathbf{A}}_{f} \widetilde{\mathbf{D}}_{f}^{-\frac{1}{2}} \mathbf{H}_{f}^{(l)} \mathbf{W}_{f}^{(l)}\right),\end{align*}

where $\mathbf{W}_{f}^{(l)}$ represents the weight matrix of the $l$th layer in feature convolution, with initial condition $\mathbf{H}_{f}^{(0)} = \mathbf{X}$. The normalized feature adjacency matrix is defined as $\widetilde{\mathbf{A}}_{f} = \mathbf{A}_{f} + \mathbf{I}$, where $\widetilde{\mathbf{D}}_{f}$ is the corresponding diagonal degree matrix.

#### Co-convolution

Gene expression and spatial distribution exhibit inherent correlation, requiring simultaneous consideration to extract commonalities between them. A parameter-sharing strategy extracts co-embeddings of gene expression and spatial distribution [32]. The propagation rules are defined as


(5) \begin{align*} & \mathbf{H}_{sc}^{(l+1)} = \mathrm{ReLU}\left(\widetilde{\mathbf{D}}_{s}^{-\frac{1}{2}} \widetilde{\mathbf{A}}_{s} \widetilde{\mathbf{D}}_{s}^{-\frac{1}{2}} \mathbf{H}_{sc}^{(l)} \mathbf{W}_{c}^{(l)}\right) \end{align*}

(6) \begin{align*} & \mathbf{H}_{fc}^{(l+1)} = \mathrm{ReLU}\left(\widetilde{\mathbf{D}}_{f}^{-\frac{1}{2}} \widetilde{\mathbf{A}}_{f} \widetilde{\mathbf{D}}_{f}^{-\frac{1}{2}} \mathbf{H}_{fc}^{(l)} \mathbf{W}_{c}^{(l)}\right), \end{align*}

where $\mathbf{W}_{c}^{(l)}$ is the shared weight matrix of the $l$th layer, $\mathbf{H}_{sc}^{(l)}$ and $\mathbf{H}_{fc}^{(l)}$ are the $l$th layer embeddings from spatial and feature graphs, respectively, with initial condition $\mathbf{H}_{sc}^{(0)} = \mathbf{H}_{fc}^{(0)} = \mathbf{X}$. The co-embedding $\mathbf{H}_{c}$ is obtained by averaging the spatial and feature co-embeddings:


(7) \begin{align*}& \mathbf{H}_{c}^{(l)} = \frac{\mathbf{H}_{sc}^{(l)} + \mathbf{H}_{fc}^{(l)}}{2}\end{align*}

To enhance commonality between $\mathbf{H}_{sc}$ and $\mathbf{H}_{fc}$, a consistency constraint is applied:


(8) \begin{align*}& \mathcal{L}_{con} = \|\widetilde{\mathbf{H}}_{sc}\widetilde{\mathbf{H}}_{sc}^{T} - \widetilde{\mathbf{H}}_{fc}\widetilde{\mathbf{H}}_{fc}^{T}\|_{2}^{2},\end{align*}

where $\widetilde{\mathbf{H}}_{sc}$ and $\widetilde{\mathbf{H}}_{fc}$ are the normalized matrices of $\mathbf{H}_{sc}$ and $\mathbf{H}_{fc}$.

#### Attention mechanism

In spatial domain identification tasks, gene expression, spatial information, and their common representation contribute differently to the results. An attention mechanism adaptively learns the importance of latent embeddings. For the co-embedding $\mathbf{H}_{c}$, a nonlinear transformation and shared attention vector $\mathbf{W}_{co}$ are applied to obtain attention coefficient $a_{c}$:


(9) \begin{align*}& a_{c} = \mathrm{softmax}(\mathbf{W}_{co} \cdot \sigma(\mathbf{W}\mathbf{H}_{c} + \mathbf{b})),\end{align*}

where $\sigma $ denotes tanh activation, $\mathbf{W}$ is the trainable weight matrix, and $\mathbf{b}$ is the bias vector. Similarly, attention coefficients $a_{s}$ and $a_{f}$ are computed for embeddings $\mathbf{H}_{s}$ and $\mathbf{H}_{f}$. The final embedding $\mathbf{H}$ is obtained by combining these embeddings:


(10) \begin{align*}& \mathbf{H} = F(a_{s} \cdot \mathbf{H}_{s} + a_{f} \cdot \mathbf{H}_{f} + a_{c} \cdot \mathbf{H}_{c}),\end{align*}

where $F$ represents a single linear layer that learns highly variable features of latent representations.

### Multi-view contrastive learning module

Recent advances in graph contrastive learning, particularly DGI, have demonstrated significant potential in unsupervised representation learning by maximizing mutual information between local node-level and global graph-level representations. However, these methods are primarily designed for general graph data and fail to address the unique challenges of ST data, including dual-graph relationships, spatial coherence constraints, and the complex interplay between molecular profiles and tissue architecture. To address these limitations, we propose a multi-view contrastive learning framework that extends graph-based mutual information maximization to the dual-graph setting of spatial transcriptomics. Unlike traditional single-graph approaches that operate on homogeneous graph structures, our method simultaneously considers both spatial proximity relationships and gene expression similarity patterns through coordinated contrastive objectives. This multi-view framework enables the model to capture the intricate biological relationships that exist between spatial organization and molecular heterogeneity in tissue samples, leading to more robust and biologically meaningful representations for spatial domain identification.

#### Spatial-aware global representation

Building upon the concept of mutual information maximization between local and global representations, we design a spatial-aware global representation that incorporates the unique characteristics of ST data. The global representation $\mathbf{s}$ is computed using an adaptive readout function:


(11) \begin{align*}& \mathbf{s} = \mathrm{SpatialReadout}(\{\mathbf{h}_{i}, \mathbf{A}_{s}, \forall i \in V\}) = \frac{\sum_{i=1}^{n} w_{i} \mathbf{h}_{i}}{\sum_{i=1}^{n} w_{i}},\end{align*}

where $w_{i}$ represents the spatial centrality weight of spot $i$, computed based on its connectivity in the spatial graph $\mathbf{A}_{s}$.

#### Dual-graph contrastive objective

Our contrastive learning framework operates on both spatial and feature graphs simultaneously, maximizing mutual information between local node representations and global tissue architecture. The discriminator network $\mathcal{D}$ is enhanced to handle multi-view inputs:


(12) \begin{align*}& \mathcal{D}(\mathbf{h}_{i}, \mathbf{s}, \mathbf{A}_{s}) = \sigma(\mathbf{h}_{i}^{T} \mathbf{W}_{spatial} \mathbf{s} + \mathrm{SpatialBias}(\mathbf{A}_{s}, i)),\end{align*}

where $\mathrm{SpatialBias}(\mathbf{A}_{s}, i)$ incorporates spatial context information for spot $i$.

#### Adaptive negative sampling

To learn robust node representations, we employ an adaptive negative sampling strategy that leverages dual-graph structures to optimize the contrastive learning process. This approach explicitly targets “hard negatives”—defined as nodes that are structurally distinct from the anchor yet exhibit high semantic similarity in the latent space. By focusing on these challenging samples, the strategy encourages the model to capture finer-grained distinctions and learn more discriminative embeddings.

Given the node embeddings $\mathbf{H}$ and adjacency matrices $\mathbf{A}_{s}$ (spatial) and $\mathbf{A}_{f}$ (feature), the procedure for selecting a negative sample for node $i$ is defined as follows:

1. Neighbor exclusion.

We first define an exclusion set $\mathcal{E}_{i}$ containing nodes that are structurally or semantically related to the anchor node $i$. This effectively removes potential positive signals from the negative candidate pool:


(13) \begin{align*}& \mathcal{E}_{i} = \{i\} \cup \{j \mid \mathbf{A}_{s}[i,j]> 0\} \cup \{j \mid \mathbf{A}_{f}[i,j] > 0\},\end{align*}

where $\mathbf{A}_{s}$ and $\mathbf{A}_{f}$ denote the spatial and feature adjacency matrices, respectively.

2. Candidate similarity computation.

For the set of valid candidates $\mathcal{C}_{i} = \{k \mid k \notin \mathcal{E}_{i}\}$, we compute the pairwise cosine similarity with the anchor node $i$:


(14) \begin{align*}& \mathrm{sim}(i, k) = \frac{\mathbf{h}_{i} \cdot \mathbf{h}_{k}}{\|\mathbf{h}_{i}\|_{2} \|\mathbf{h}_{k}\|_{2}}, \quad \forall k \in \mathcal{C}_{i}\end{align*}

3. Hard negative selection.

We select the candidate $k^{*}$ that exhibits the **maximum** similarity to node $i$ among the non-neighbors. This “hard negative” forces the discriminator to learn finer-grained features to distinguish distinct domains:


(15) \begin{align*}& k^{*} = \underset{k \in \mathcal{C}_{i}}{\arg\max} \ \mathrm{sim}(i, k)\end{align*}

If the candidate set $\mathcal{C}_{i}$ is empty, we fallback to random sampling. The final negative embedding matrix $\widetilde{\mathbf{H}}$ is constructed by assigning $\widetilde{\mathbf{h}}_{i} = \mathbf{h}_{k^{*}}$.

#### DGI objective

The final contrastive objective combines information from both graph views and incorporates spatial regularization:


(16) \begin{align*}& \begin{aligned} \mathcal{L}_{DGI} = &-\frac{1}{n} \sum_{i=1}^{n} \left[ \log \mathcal{D}(\mathbf{h}_{i}, \mathbf{s}, \mathbf{A}_{s}) + \log (1 - \mathcal{D}(\mathbf{h}_{neg,i}, \mathbf{s}, \mathbf{A}_{s})) \right] \\ &+ \lambda_{spatial} \sum_{(i,j) \in \mathcal{E}_{spatial}} \|\mathbf{h}_{i} - \mathbf{h}_{j}\|_{2}^{2} \end{aligned}\end{align*}

This formulation ensures that the learned representations capture both local molecular signatures and global tissue organization patterns, making it particularly suitable for spatial domain identification tasks.

#### Integration with multi-view architecture

The contrastive learning module is seamlessly integrated with our multi-view GCN encoder, where the contrastive loss operates on the attention-weighted embeddings:


(17) \begin{align*}& \mathbf{h}_{contrast} = \mathrm{MLP}(a_{s} \cdot \mathbf{H}_{s} + a_{f} \cdot \mathbf{H}_{f} + a_{c} \cdot \mathbf{H}_{c})\end{align*}

This integration allows the contrastive learning to benefit from the rich multi-view representations while providing feedback to improve the attention mechanism.

### ZINB decoder

ST data exhibit high sparsity and overdispersion [38]. Thus, we employ a ZINB [37] decoder to model the gene expression distribution. The ZINB decoder consists of three neural networks that output parameters for the ZINB distribution:


(18) \begin{align*}& \begin{aligned} \pi_{i} &= \mathrm{Sigmoid}(f_{\pi}(\mathbf{h}_{i})) \\ \mu_{i} &= \mathrm{MeanAct}(f_{\mu}(\mathbf{h}_{i})) \\ \theta_{i} &= \mathrm{DispAct}(f_{\theta}(\mathbf{h}_{i})) \end{aligned},\end{align*}

where $f_\pi $, $f_\mu $, and $f_\theta $ are single-layer neural networks, MeanAct and DispAct are activation functions ensuring positive outputs:


(19) \begin{align*}& \begin{aligned} \mathrm{MeanAct}(x) &= \mathrm{Clamp}(e^{x}, 10^{-5}, 10^{6}) \\ \mathrm{DispAct}(x) &= \mathrm{Clamp}(\mathrm{Softplus}(x), 10^{-4}, 10^{4}) \end{aligned}\end{align*}

The ZINB loss function is defined as


(20) \begin{align*}& \mathcal{L}_{ZINB} = - \sum_{i=1}^{n} \sum_{g=1}^{d} \log P_{ZINB} \left( x_{ig} \mid \pi_{ig}, \mu_{ig}, \theta_{ig} \right)\end{align*}

### Spatial regularization

To preserve spatial coherence in the learned embeddings, we incorporate a spatial regularization term that encourages similar representations for spatially adjacent spots while pushing apart nonadjacent spots:


(21) \begin{align*}& \mathcal{L}_{reg} = \sum_{(i,j) \in \mathcal{E}_{spatial}} \frac{\lVert \mathbf{h}_{i} - \mathbf{h}_{j} \rVert_{2}^{2}}{2} - \lambda \sum_{(i,k) \in \mathcal{E}_{neg}} \frac{\lVert \mathbf{h}_{i} - \mathbf{h}_{k} \rVert_{2}^{2}}{2},\end{align*}

where $\mathcal{E}_{spatial}$ denotes positive pairs (spatial neighbors), $\mathcal{E}{neg}$ denotes negative pairs (non-neighbors), and $\lambda $ is a balancing parameter.

### Joint optimization

The model is trained end-to-end by jointly optimizing multiple objectives:


(22) \begin{align*}& \mathcal{L}_{total} = \alpha \mathcal{L}_{ZINB} + \beta \mathcal{L}_{con} + \gamma \mathcal{L}_{reg} + \delta \mathcal{L}_{DGI},\end{align*}

where $\alpha $, $\beta $, $\gamma $, and $\delta $ are hyperparameters controlling the relative importance of each loss component. We set $\alpha = 10.0$, $\beta = 0.1$, $\gamma = 0.1$, and $\delta = 0.15$ based on validation experiments.

### Spatial domain identification

After training, the learned embeddings $\mathbf{H}$ are used for spatial domain identification through K-means clustering:


(23) \begin{align*}& C = \mathrm{KMeans}\left(H, K\right),\end{align*}

where $K$ is the number of spatial domains(clusters), typically determined based on prior biological knowledge or through unsupervised selection methods.

### Evaluation metrics

The metrics of election for clustering include Adjusted Rand Index (ARI). ARI adjusts the Rand Index to account for chance agreement, providing a measure that ranges from −1 to 1: values near 1 indicate strong concordance between clustering solutions, values around 0 correspond to random agreement, and negative values indicate less agreement than expected by chance.

### Data preprocessing

We evaluate SpatialDG on four public spatial transcriptomics datasets ecompassing different technologies and tissue types:

**Human DLPFC Dataset:** a human brain dorsolateral prefrontal cortex (DLPFC) dataset with 12 tissue slices acquired with 10x Visium [39]. The number of spots in each slice ranged from 3460 to 4789, with 33 538 genes captured. Each slice was manually annotated to contain five to seven regions, namely the DLPFC layers and white matter.

**Human Breast Cancer Dataset:** a 10x Visium dataset [40] annotated by Fu *et al*. [41] into 20 regions representing four main morphological categories: ductal carcinoma *in situ*/lobular carcinoma *in situ* (DCIS/LCIS), invasive ductal carcinoma (IDC), healthy tissue, and tumor edge regions with low malignancy. These annotated areas served as the baseline for the evaluation of clustering. This dataset consisted of 3798 spots and 36 601 genes.

**Mouse Visual Cortex Dataset:** obtained using STARmap technology [42], this dataset provides single-cell resolution spatial mapping of the mouse visual cortex.

**Mouse Embryo Dataset:** generated using Stereo-seq [43], this dataset captures mouse embryonic development at stage E9.5, providing high-resolution spatial gene expression during whole-embryo organogenesis.

**Mouse Olfactory Bulb Dataset:** The Slide-seqV2 mouse olfactory bulb data were profiled at a spatial resolution of 10 $\mu $m, and contain 19 285 and 20 139 spots [44]. We also downloaded publicly available the annotation atlas images from the Allen Brain Atlas website [45].

For all datasets, we selected the top 3000 highly variable genes using the Seurat v3 method implemented in Scanpy [46]. Gene expression data were library-size normalized, log-transformed, and scaled before being used as input to SpatialDG.

### Benchmark methods

In this section, we compare SpatialDG against six state-of-the-art ST analysis methods that represent different methodological approaches:

**SpaGCN** [27]: a GCN that incorporates histology information through an adaptive graph construction strategy, combining gene expression and spatial location with optional histological images.

**STAGATE** [28]: an adaptive graph attention auto-encoder that employs an attention mechanism to adaptively learn the similarity between neighboring spots, enabling effective integration of spatial information.

**GraphST** [35]: a graph self-supervised contrastive learning framework that combines GNNs with self-supervised learning to jointly model gene expression and spatial location.

**SEDR** [31]: a deep learning method that uses a variational graph auto-encoder to embed both gene expression and spatial information into a latent representation for spatial domain detection.

**Spatial-MGCN** [36]: a spatial clustering algorithm utilizing a multi-view GCN encoder to extract gene expression information, spatial information, and their combinations.

**stLearn** [10]: a method for spatial domain identification by combining gene expression, spatial information, and morphological features obtained from histological images.

All baseline methods were executed using their default parameters as specified in the original publications, with the exception of the number of spatial domains, which was set consistently across all methods based on ground truth annotations (seven for DLPFC brain tissue) or prior biological knowledge (25 for mouse embryo). For methods requiring additional hyperparameter tuning, we used the recommended strategies from their respective documentations. All experiments were conducted on the same computational infrastructure to ensure fair comparison.

### Implementation details

In this paper, SpatialDG is implemented in PyTorch 2.5.1. The model architecture consists of a two-layer GCN encoder with hidden dimensions of [128, 64]. A dropout rate of 0.1 is applied after the first GCN layer to prevent overfitting. We optimize the model using the Adam optimizer with a learning rate of $10^{-3}$ and a weight decay of $10^{-5}$. The model is trained for 200 epochs with an early stopping mechanism based on the reconstruction loss.

Regarding hyperparameter initialization, we strictly aligned our settings with the protocols established in STAGATE [28] to ensure topological consistency across platforms. Specifically, for the DLPFC, Human Breast Cancer (HBC), and Mouse Embryo datasets, the spatial neighbor radius is set to $r=550$. This threshold is physically grounded, as it strictly covers the central spot and its six immediate neighbors in the standard Visium hexagonal grid, ensuring an accurate representation of local tissue topology. For the high-resolution Mouse Olfactory Bulb dataset (Slide-seqV2), we adjusted the radius to $r=50$. This adaptive setting ensures that the constructed spatial graphs capture biologically relevant local neighborhoods regardless of the varying coordinate systems and spot resolutions.

The number of nearest neighbors for the feature graph is set to $k=15$, consistent with the settings in Spatial-MGCN [36], to balance local semantic smoothing with computational efficiency.

All experiments were performed on a workstation powered by an MacBook M4 Pro chip (14-core CPU, 20-core GPU, 16-core Neural Engine) with 24 GB of Unified Memory. SpatialDG demonstrates remarkable computational efficiency and low memory requirements across datasets of varying scales. For standard benchmarks, the model converges rapidly: the DLPFC dataset ( 3600 spots) required only 118.4 s with a peak memory usage of 1.10 GB, while the Human Breast Cancer (HBC) dataset completed training in 105.3 s using just 0.76 GB of memory. Furthermore, the framework exhibits strong scalability on large-scale datasets. The Mouse Embryo dataset required 1831 s ($\sim $30 min) with 1.44 GB peak memory, and the high-resolution Mouse Olfactory Bulb dataset completed in 2115 s ($\sim $35 mins) consuming only 2.06 GB of memory. These results confirm that SpatialDG is lightweight and easily deployable on standard consumer hardware.

## Results

### SpatialDG enhances detection of stratified architectural patterns in human dorsolateral prefrontal cortex tissue

**Figure 2:**
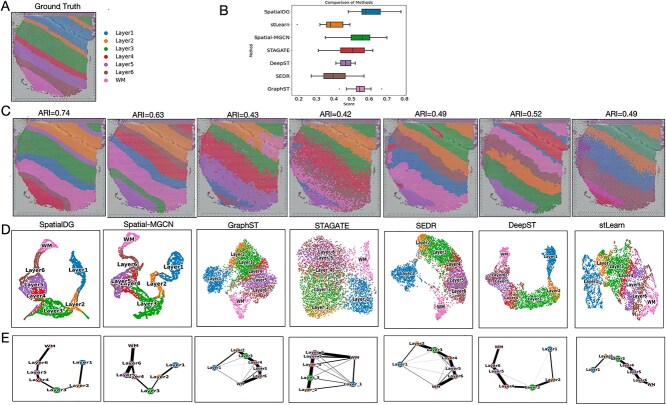
**SpatialDG outperforms state-of-the-art methods in the DLPFC dataset**: (A) Manually annotated brain regions (ground truth) of a representative slice (151507), and (B) overall performance of eight different methods across all the 12 slices comprised in the dataset; (C) unsupervised domain identification results on slice 151507; (D) UMAP visualization results of the embeddings from 8 different methods on slice 151507 and, (E) inferred trajectories on slice 151507.

The spatial architecture of the brain is closely linked to its function, a relationship particularly evident in the layered organization of the human cerebral cortex [47]. To investigate the ability of SpatialDG to capture the arrangement of brain structures, we made use of a 10x Visium dataset comprising 12 DLPFC sections [39]. Manually annotated, ground truth layered structures are shown in Fig. 2A.

We initially compared the ARI levels of various methods across 12 slices of the DLPFC dataset Fig. 2B, Supplementary Fig.1). SpatialDG achieved the highest median ARI score(0.60), significantly outperforming existing methods, where the second-best (Spatial-MGCN) obtained a median ARI of $\sim $0.55 and TAGATE reached $\sim $0.5. Traditional methods such as stLearn and SEDR showed relatively lower performance with median ARI scores of $\sim $0.4 and 0.35, respectively, and DeepST and GraphST demonstrated intermediate performance levels. Notably, the results from STAGATE and Spatial-MGCN show differences in the ARI across different slices, suggesting that these methods are sensitive to the variation of domain patterns across sections, while SpatialDG maintains more consistent performance across all tissue sections (Supplementary Table 1).

We further investigated cross-section results by conducting a detailed analysis for each slice (a representative slice is shown as an example in Fig. 2C–E). Results show the inconsistency and sources of bias of the state-of-the-art methods, with DeepST and stLearn struggling with rough segmentation between layers and producing fragmented clustering patterns that fail to capture the continuous cortical organization, SEDR exhibiting issues with erroneous region identification, particularly in distinguishing between adjacent cortical layers, and GraphST and STAGATE showing significant biases in identifying the precise boundaries between distinct domains, often resulting in over-segmentation or irregular domain shapes. In this specific case, SpatialDG demonstrates exceptional domain identification results, accurately reconstructing the laminar cortical structure with clear layer-specific boundaries that closely match the ground truth annotation. We further utilize UMAP for low-dimensional visualization analysis of the results obtained from different methods (Fig. 2D) to verify whether the embeddings can accurately encompass information on regional arrangement and boundaries. The analysis reveals that SpatialDG produces the most biologically plausible connectivity pattern, with each domain connecting primarily to its anatomically adjacent layers. Spatial-MGCN also maintains reasonable adjacency relationships, effectively separating different cortical domains. In contrast, GraphST, STAGATE, and other methods exhibit noticeable issues in domain connectivity, showing either oversimplified or overly complex adjacency patterns.

As a complementary approach, we performed trajectory inference using the PAGA algorithm [48] for all the methods (Fig. 2E). The PAGA graphs indicate that SpatialDG, Spatial-MGCN, STAGATE, and DeepST all perform well in predicting trajectory between adjacent layers, while GraphST, SEDR, and stLearn exhibit either oversimplified linear connections or inappropriately complex adjacency relationships that do not accurately represent the spatial organization of cortical domains. Once again, SpatialDG demonstrates the most accurate connectivity, with each domain primarily connecting to its anatomically adjacent layers in a manner consistent with cortical organization.

### SpatialDG imputes gene expression to further decipher spatial patterns

**Figure 3:**
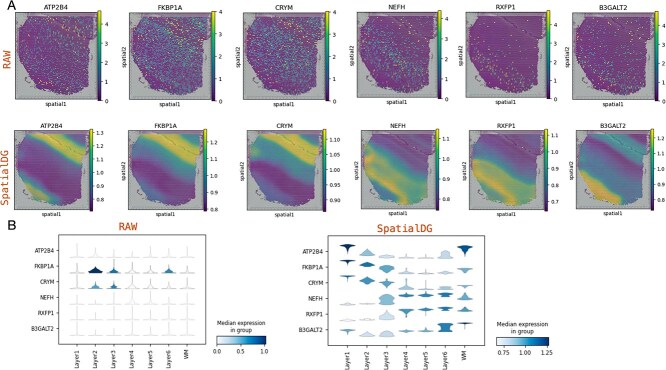
**SpatialDG enhances spatial gene expression profiles and spatial structural characterization**: (A) Spatial representation of layer-specific marker genes before and after data enhancement by SpatialDG, and (B) gene expression level before and after data enhancement.

In spatial transcriptomics, the analysis of domain-specific genes is crucial to understand tissue architecture and function. However, identifying domain-specific genes remains challenging due to substantial noise in gene expression profiles generated by spatial transcriptomic techniques, including dropout events and technical artifacts. Therefore, robust feature learning methods should effectively separate irrelevant interference factors from raw data while preserving key tissue distribution information. As shown in Fig. 3A, we compared the expression of six original layer-marker genes (ATP2B4, FKBP1A, CRYM, NEFH, RXFP1, B3GALT2) [49] in DLPFC slice 151507 with the expression patterns reconstructed based on SpatialDG. The raw data exhibit significant noise and dropout events, making it difficult to discern clear spatial expression boundaries between cortical layers. In contrast, after enhancement with SpatialDG, the same genes demonstrate dramatically improved spatial organization and expression clarity. At quantitative level (Fig. 3B), the raw data show minimal expression variation across layers for most genes, with FKBP1A and CRYM being the only genes showing detectable layer-specific patterns. In stark contrast, SpatialDG-enhanced data reveal distinct expression distributions for all analyzed genes across different cortical layers. For instance, ATP2B4 shows clear bimodal expression with peaks in Layers 1 and 6, FKBP1A exhibits highest expression in Layers 2 and 3, and NEFH demonstrates a gradient increase from superficial to deep layers, in line with their functions within the cortical architecture [50–52]. These results demonstrate that SpatialDG not only reduces technical noise but also reveals biologically meaningful spatial gene expression patterns otherwise masked in the raw data. The enhanced expression profiles show improved spatial coherence and layer-specific organization, enabling more accurate identification of domain-specific markers and better understanding of the molecular architecture underlying cortical lamination. This enhancement capability is particularly valuable for downstream analyses such as spatial domain characterization, pathway analysis, and understanding spatial gene regulatory networks.

### SpatialDG accurately identifies tumor region in human breast cancer

**Figure 4:**
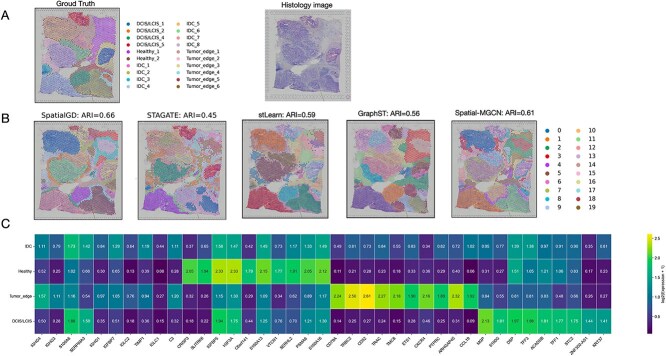
**Comparative analysis of spatial domain identification methods in breast cancer**: (A) Ground truth annotation and corresponding histology image, with annotations spanning IDC, ductal/lobular carcinoma *in situ* (DCIS/LCIS), healthy tissue, and tumor edge regions; (B) spatial domain predictions by different methods with corresponding ARI scores: SpatialDG, Spatial-MGCN, stLearn, GraphST, and STAGATE; (C) differential gene expression heatmap across identified spatial domains, showing domain-specific molecular signatures, from SpatialDG.

Breast cancer ranks among the leading cancer types globally [53], and is characterized by high inter- and intra-sample variability at the genetic, molecular, and clinical level. This makes breast cancer datasets an optimal testbed to validate SpatialDG’s performance in heterogeneous tissue architectures. This dataset was obtained from the 10x Genomics Visium open access platform (https://support.10xgenomics.com/) and used to conduct an in-depth analysis of the breast cancer microenvironment (Fig. 4), comparing with existing spatial analysis methods. Figure 4A shows the ground truth annotation of the breast cancer dataset, which contains multiple distinct regions including healthy tissue, IDC, ductal carcinoma *in situ*/lobular carcinoma *in situ* (DCIS/LCIS), and tumor edge regions. The corresponding histology image reveals the complex architectural organization of breast cancer tissue, with both areas of clear morphological distinctions between different tissue types and areas of admixture and proximity. Figure 4B shows spatial domain identification results from different methods. SpatialDG achieved again the highest ARI score of 0.66, significantly outperforming other methods including Spatial-MGCN (ARI=0.61), stLearn (ARI=0.59), GraphST (ARI=0.56), and STAGATE (ARI=0.45). Notably, while STAGATE can identify some major tissue regions, it exhibits significant cluster mixing and substantial presence of outliers, particularly in the boundary regions between healthy and cancerous tissue. Meanwhile, stLearn produces relatively smoother results but fails to accurately delineate healthy tissue regions, often dividing them into fragmented small clusters. GraphST shows intermediate performance but struggles with precise boundary identification between different cancer subtypes. In contrast, SpatialDG not only identifies tissue structures highly consistent with manual annotation, but also achieves the smoothest tissue boundaries with minimal outliers. Figure 4C shows the expression patterns of domain-specific marker genes across all identified regions. Gene imputation by SpatialDG unmasks significant molecular heterogeneity among different tissue types, which is again in line with biological cues. For example, IDC regions show elevated expression of signature genes such as IGFBP7 [54] (Z=1.29) and TIMP1 [55] (Z=1.19), along with IGHG4, IGHG3 [56], and S100A6 [57], which have been previously associated with invasive carcinoma characteristics. Healthy tissue areas show high expression of IGFBP5 [58] (Z=2.33), and H3F3A [59] (Z=2.33), along with SLITRK6 [60], reflecting normal mammary epithelial function. Tumor edge regions are characterized by immune-related genes including CD79A [61] (Z=2.24), TRBC2 [62] (Z=2.50), CD52 [63] (Z=2.61), and CXCR4 [64], suggesting active immune infiltration and tumor–host interactions. Finally, DCIS/LCIS areas show distinct expression patterns of S100G [65] (Z=1.81) and TFF3 [66] (Z=1.98), along with MGP [67], DSP [68], and SERPINA3 [69], reflecting preinvasive carcinoma characteristics. Taken together, these findings demonstrate the superior performance of SpatialDG in clustering heterogeneous tissues while highlighting functional and regulatory differences between different tumor tissue subtypes and providing clearly separated gene expression profiles across spatially defined domains.

### SpatialDG reveals fine-grained tissue structures in mouse embryo Stereo-seq data

In this section, we utilized a mouse embryonic (E9.5 stage) dataset obtained via Stereo-seq technology, comprising 5913 compartments and 25 568 genes [43], to test SpatialDG performance on a different methodological approach (DNA nanoballs) with dramatically higher resolution than Visium. Tissue domain annotation, taken as the ground truth, are taken from the original study where SCANPY’s [46] Leiden clustering algorithm was used to process neighborhood graphs constructed on spatial proximity and transcriptomic similarity, annotating the resulting clusters according to differentially expressed gene pairs.

**Figure 5:**
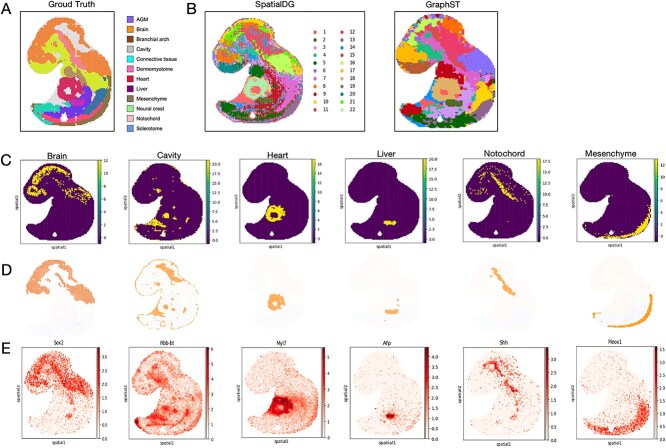
**SpatialDG enhances biological structure identification at high resolution**: (A) Organ annotations (ground truth) for the E9.5 mouse embryo, and (B) clustering results of SpatialDG and GraphST on the E9.5 mouse embryo data; (C) visualization of selected spatial domains identified by SpatialDG, showing high correspondence with tissue type annotation results (D) and accurate marker gene identification (E).

The original annotation contained reference clusters as in Fig. 5A. We set the cluster count to 22 in our tests to achieve higher resolution tissue segmentation. Compared with the ground truth, the output from GraphST identified numerous clusters that were less clearly delineated and showed limited correspondence with the annotated tissue architecture (Fig. 5B). In contrast, SpatialDG demonstrated higher concordance with annotated regions (Fig. 5B), successfully identifying major tissue types that corresponded well with the original tissue architecture (Fig. 5C and D). SpatialDG effectively captured the spatial organization of key developmental genes within their corresponding structures, with brain regions highly correlating with Sox2 [70], cavity with Hbb-bt [71], heart tissue with Myl7 [72], liver with Afp [73], notochord with Shh [74], and various mesenchymal tissues with Meox1 [75]. More importantly, gene patterns imputed by SpatialDG more closely resemble actual *in situ* staining for the same gene, e.g. Sox2 in the developing brain [76] or Myl17 in the heart precursors [77].

### SpatialDG identifies the laminar organization in the mouse olfactory bulb tissue Slide-seqV2 data

**Figure 6:**
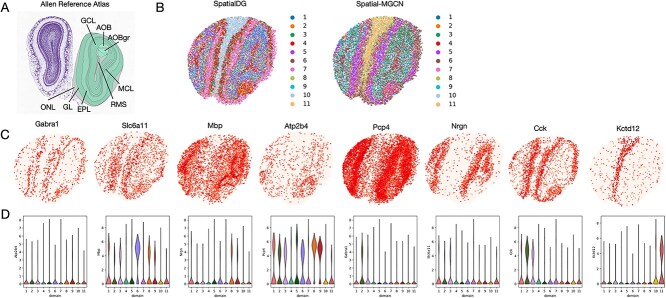
**Spatial domain identification and biological validation in the mouse olfactory bulb dataset by Slide-seqV2**: (A) Laminar organization of mouse olfactory bulb annotated by the Allen Reference Atlas; (B) comparison of spatial domains identified by SpatialDG and Spatial-MGCN, SEDR; (C) spatial expression heatmaps of eight representative layer-specific marker genes (Gabra1, Slc6a11, Mbp, Atp2b4, Pcp4, Nrgn, Cck, Kctd12), with color intensity indicating expression levels; (D) violin plot of domain-marker gene expressions in derived clusters by SpatialDG.

We further applied SpatialDG to analyze a mouse olfactory bulb dataset generated using Slide-seq V2. The spatial domains identified by SpatialDG exhibited strong alignment with the annotated structures in the Allen Reference Atlas (Fig. 6A). Specifically, SpatialDG successfully delineated multiple anatomically relevant regions, including the granule cell layer, accessory olfactory bulb (AOB), its granular layer (AOBgr), mitral cell layer (MCL), olfactory nerve layer, glomerular layer (GL), external plexiform layer, and the rostral migratory stream.

Notably, compared with the fragmented and discontinuous clusters generated by baseline methods Spatial-MGCN (Fig. 6B), SpatialDG produced spatially continuous and smoothly bounded domains that closely reflect the fine-grained laminar organization of the olfactory bulb. These domains were further validated by known layer- and region-specific marker genes (Fig. 6C). For example, Gabra1, a marker for mitral cells, was enriched in the MCL domain, while Slc6a11, associated with inhibitory neuronal function, showed prominent expression in granular and GLs. Quantification of expression patterns for these and additional established markers, including Gabra1, Slc6a11, Mbp, Atp2b4, Pcp4, Nrgn, Cck, and Kctd12, confirmed their specific enrichment in corresponding SpatialDG identified domains (Fig. 6D), further supporting the biological relevance of the revealed laminar structure.

These results demonstrate that SpatialDG can accurately identify spatially coherent tissue structures and capture their molecular signatures, even in complex neural architectures such as the olfactory bulb. The method’s ability to align with established anatomical atlases and recover biologically meaningful gene expression patterns underscores its utility for high-resolution spatial domain discovery in spatially resolved transcriptomics data.

### Ablation study

To evaluate the effectiveness of each component in SpatialDG, we conducted systematic ablation experiments on the DLPFC and Breast Cancer datasets. We sequentially removed key modules, including (1) the dual-graph architecture, (2) the DGI module, (3) the consistency loss, and (4) the ZINB reconstruction loss. By comparing the performance of the full model with each ablated variant, we quantified the contribution of each component to spatial domain identification.

Table 1 presents the performance comparison between the full model and the ablated variants. The complete SpatialDG framework achieved an ARI of **0.60** on the DLPFC dataset and **0.66** on the Breast Cancer dataset, significantly outperforming all ablated versions. The specific contributions of each module are analyzed below:

1. **Importance of dual-graph architecture:** Removing the dual-graph structure (*w/o Dual-graph*) led to a consistent performance decline: ARI decreased by **11.3%** on DLPFC and by **22.7%** on the Breast Cancer dataset. The sharper drop in the cancer dataset demonstrates that integrating spatial proximity with gene expression similarity is particularly crucial for dissecting complex, heterogeneous tumor microenvironments, where a single spatial graph is insufficient.
2. **Contribution of contrastive learning:** Removing the DGI module (*w/o DGI*) reduced the ARI by **14.1%** on DLPFC and **10.6%** on Breast Cancer data. This consistent drop across tissues indicates that the Deep Graph Infomax mechanism effectively enhances embedding discriminability. By maximizing the mutual information between local and global representations, the model captures distinctive features critical for separating both laminar (brain) and irregular (tumor) domains.
3. **Role of consistency constraint:** Eliminating the consistency loss (*w/o Consistency*) decreased the ARI by **10.6%** (DLPFC) and **13.6%** (Breast Cancer). This validates the necessity of dual-graph embedding alignment. The consistency constraint ensures semantic coherence between features extracted from the two graph views, preventing fragmentation in the representation space regardless of the tissue type.
4. **Criticality of ZINB reconstruction:** Removing the ZINB loss (*w/o ZINB*) caused the most drastic performance drop on DLPFC (−35.2%) and a significant decline on Breast Cancer data (−18.2%). This demonstrates that generative reconstruction is indispensable for modeling scRNA-seq data properties. The ZINB distribution effectively handles the zero-inflation and overdispersion characteristics inherent in spatial transcriptomics platforms, providing a robust foundation for feature learning.

**Table 1:** Performance comparison of SpatialDG and its ablated variants on the DLPFC and Breast Cancer datasets

Method	ARI(DLPFC)	ARI(BC)
SpatialDG	**0.60**	**0.66**
w/o Dual-graph	0.53	0.51
w/o DGI	0.52	0.59
w/o Consistency	0.54	0.57
w/o ZINB	0.39	0.54

**Note:**  *w/o Dual-graph*: spatial graph $G_{s}$ only; *w/o DGI*: removal of Deep Graph Infomax; *w/o Consistency*: removal of embedding alignment; *w/o ZINB*: removal of ZINB reconstruction loss.

## Discussion and conclusion

In this paper, we present SpatialDG, a novel dual-GNN framework that significantly advances the field of unsupervised spatial clustering by effectively integrating molecular and spatial information through a unified contrastive learning framework. Results show that SpatialDG outperforms state-of-the-art methods in terms of clustering accuracy and identifying biologically relevant domains. In the human DLPFC dataset, SpatialDG achieved the highest median ARI score (0.60) while successfully reconstructing the cortical laminar organization with biologically meaningful and layer-specific boundaries. In breast cancer, likewise, SpatialDG achieved highest accuracy (ARI=0.66), effectively distinguishing between healthy tissue, IDC and other cancer subtypes, while maintaining smooth tissue boundaries with minimal outliers. Finally, the high-resolution Stereo-Seq mouse embryo results further validated SpatialDG capacity to capture complex developmental tissue organization, successfully identifying major organ systems with appropriate spatial coherence and much finer details recapitulating the actual biological truth than other methods.

The superior performance of SpatialDG can be attributed to two mechanistic advantages verified by our ablation study. First, the dual-graph architecture mitigates the over-smoothing problem common in methods relying solely on spatial proximity by supplementing spatial information with semantic connections via the feature graph. This allows the model to differentiate spatially adjacent but biologically distinct spots, such as sharp tumor boundaries. Second, unlike simple reconstruction losses that focus primarily on data fidelity, the contrastive learning module enforces class separability in the latent space. This discriminative power is critical for disambiguating subtle transition zones in heterogeneous tissues, enabling the precise delineation of complex interfaces that reconstruction alone might blur.

Beyond spatial domain identification, SpatialDG demonstrates remarkable capability in gene expression imputation and noise reduction. The analysis of layer-specific marker genes in DLPFC tissue revealed that while raw data exhibited substantial noise and dropout events, SpatialDG-enhanced data showed clear spatial expression boundaries and biologically meaningful layer-specific patterns. This enhancement capability extends the method’s utility to downstream analyses including spatial domain characterization, pathway analysis, and spatial gene regulatory network inference.

SpatialDG demonstrates robust performance but has limitations. First, while achieving the highest median ARI, certain baseline methods occasionally outperformed it on individual tissue slices (Supplementary Table 1), typically in cases of extreme noise or highly ambiguous transitional regions where the contrastive objective’s focus on discriminative separation may under-smooth local heterogeneity. Second, the dual-graph architecture entails greater computational cost than single-graph methods, which may challenge scaling to future ultra-high-resolution datasets. Third, although validated across multiple platforms, fixed graph-construction hyperparameters may not optimally adapt to all tissue geometries (e.g. infiltrative tumor margins), and broader testing on imaging-based technologies (e.g. MERFISH) remains valuable.

Future work will focus on adaptive graph construction, integration of lightweight GNNs for scalability, and leveraging single-cell atlases for semi-supervised refinement. Extending the framework to multi-sample and 3D spatial transcriptomics will further enhance its utility. These directions, informed by the current limitations, will evolve SpatialDG into a more versatile and efficient tool for the community.

**Key Points**

- SpatialDG learns unsupervised tissue representations by dual-graph modeling.
- A dual-view contrastive objective maximizes the agreement between local and global embeddings, enhancing representation quality.
- Integration of a zero-inflated negative binomial (ZINB) loss models the count-based and sparse nature of transcriptomic data, improving robustness to technical noise.
- SpatialDG outperforms existing unsupervised methods in identifying spatially coherent and biologically meaningful tissue domains across multiple healthy and cancer datasets.

## Supplementary Material

Supplement_bbag145

## Data Availability

All the datasets used in this paper are open access and available as (1) The LIBD human dorsolateral prefrontal cortex (DLPFC) dataset http://spatial.libd.org/spatialLIBD; (2) 10x Visium spatial transcriptomics dataset of human breast cancer https://support.10xgenomics.com/spatial-gene-expression/datasets/1.1.0/V1_Breast_Cancer_Block_A_Section_1; (3) Mouse embryo Stereo-seq data https://db.cngb.org/stomics/mosta/; (4) Slide-seqV2 datasets5 are available at the Broad Institute Single Cell Portal at https://singlecell.broadinstitute.org/single_cell/study/SCP815/highly-sensitive-spatial-transcriptomics-at-near-cellular-resolution-with-slide-seqv2#study-summary. The code of SpatialDG can be downloaded from https://github.com/Izzilab/SpatialDG.

## References

[1] Rao A, Barkley D, França GS et al. Exploring tissue architecture using spatial transcriptomics. *Nature* 2021;596:211–20. 10.1038/s41586-021-03634-9 34381231 PMC8475179

[2] Asp M, Bergenstråhle J, Lundeberg J. Spatially resolved transcriptomes-next generation tools for tissue exploration. *BioEssays* 2020;42:e1900221. 10.1002/bies.201900221 32363691

[3] Satija R, Farrell JA, Gennert D et al. Spatial reconstruction of single-cell gene expression data. *Nat Biotechnol* 2015;33:495–502. 10.1038/nbt.3192 25867923 PMC4430369

[4] Hartigan JA, Wong MA. Algorithm as 136: a k-means clustering algorithm. *J R Stat Soc Ser C Appl Stat* 1979;28:100–8.

[5] Blondel VD, Guillaume J-L, Lambiotte R et al. Fast unfolding of communities in large networks. *J Stat Mech Theory Exp* 2008;2008. 10.1088/1742-5468/2008/10/P10008

[6] Abdi H, Williams LJ. Principal component analysis. *Wiley Interdiscip Rev Comput Stat* 2010;2:433–59. 10.1002/wics.101

[7] van der Maaten , Hinton G. Visualizing data using t-SNE. *J Mach Learn Res* 2008;9:2579–605.

[8] McInnes L, Healy J, Melville J. UMAP: uniform manifold approximation and projection for dimension reduction. arXiv preprint arXiv:1802.03426. 2018.

[9] Song Q, Jing S. DSTG: deconvoluting spatial transcriptomics data through graph-based artificial intelligence. *Brief Bioinform* 2021;22:1–13. 10.1093/bib/bbaa414 PMC8425268 33480403

[10] Pham D, Tan X, Xu J et al. stLearn: integrating spatial location, tissue morphology and gene expression to find cell types, cell-cell interactions and spatial trajectories within undissociated tissues. biorxiv. 2020;2020.

[11] Dries R, Zhu Q, Dong R et al. Giotto: a toolbox for integrative analysis and visualization of spatial expression data. *Genome Biol* 2021;22:78. 33685491 10.1186/s13059-021-02286-2 PMC7938609

[12] Zhang Y, Brady M, Smith S. Segmentation of brain MR images through a hidden Markov random field model and the expectation-maximization algorithm. *IEEE Trans Med Imaging* 2002;20:45–57. 10.1109/42.906424 11293691

[13] Zhao E, Stone MR, Ren X et al. Spatial transcriptomics at subspot resolution with BayesSpace. Nat Biotechnol 2021;39:1375–84. 10.1038/s41587-021-00935-2 34083791 PMC8763026

[14] Yang B, Bao W, Wang J. Active disease-related compound identification based on capsule network. *Brief Bioinform* 2021;23:bbab462. 10.1093/bib/bbab462 PMC8690041 35057581

[15] Chen B, Li N, Bao W. CLPr_in_ML: cleft lip and palate reconstructed features with machine learning. *Current Bioinformatics* 2025;20:179–93. 10.2174/0115748936330499240909082529

[16] Bao W, Liu Y, Chen B. Oral_voting_transfer: classification of oral microorganisms’ function proteins with voting transfer model. *Front Microbiol* 2024;14:1277121. 10.3389/fmicb.2023.1277121 38384719 PMC10879614

[17] Yuan L, Zhijie X, Meng B et al. scAMZI: attention-based deep autoencoder with zero-inflated layer for clustering scRNA-seq data. *BMC Genomics* 2025;26:350. 10.1186/s12864-025-11511-2 40197174 PMC11974017

[18] Li B, Tang Z, Budhkar A et al. SpaIM: single-cell spatial transcriptomics imputation via style transfer. *Nat Commun* 2025;16:7861. 10.1038/s41467-025-63185-9 40849313 PMC12375071

[19] Zhang P, Weiqing Chen TN, Tran MZ et al. Thor: a platform for cell-level investigation of spatial transcriptomics and histology. *Nat Commun* 2025;16:7178. 40764306 10.1038/s41467-025-62593-1 PMC12325965

[20] Zonghan W, Pan S, Chen F et al. A comprehensive survey on graph neural networks. *IEEE Trans Neural Networks Learn Syst* 2020;32:4–24. 10.1109/TNNLS.2020.2978386 32217482

[21] Velickovic P, Cucurull G, Casanova A et al. Graph attention networks. *Statistics* 2017;1050:10–48550.

[22] Hamilton W, Ying Z, Leskovec J. Inductive representation learning on large graphs. *Adv Neural Inf Proces Syst* 2017;30.

[23] Felix W, Souza A, Zhang T et al. Simplifying graph convolutional networks. In:*International Conference on Machine Learning*, pp. 6861–71. Pmlr, 2019.

[24] Kipf TN . Semi-supervised classification with graph convolutional networks. arXiv preprint arXiv:1609.02907. 2016.

[25] Du J, Zhang S, Wu G et al. Topology adaptive graph convolutional networks. arXiv preprint arXiv:1710.10370. 2017.

[26] Shi Y, Huang Z, Feng S et al. Masked label prediction: unified message passing model for semi-supervised classification. arXiv preprint arXiv:2009.03509. 2020.

[27] Jian H, Li X, Coleman K et al. SpaGCN: integrating gene expression, spatial location and histology to identify spatial domains and spatially variable genes by graph convolutional network. *Nat Methods* 2021;18:1342–51. 34711970 10.1038/s41592-021-01255-8

[28] Dong K, Zhang S. Deciphering spatial domains from spatially resolved transcriptomics with an adaptive graph attention auto-encoder. *Nat Commun* 2022;13:1739. 10.1038/s41467-022-29439-6 35365632 PMC8976049

[29] Li J, Chen S, Pan X et al. Cell clustering for spatial transcriptomics data with graph neural networks. *Nat Comput Sci* 2022;2:399–408. 10.1038/s43588-022-00266-5 38177586

[30] Chang X, Jin X, Wei S et al. DeepST: identifying spatial domains in spatial transcriptomics by deep learning. *Nucleic Acids Res* 2022;50:e131–1. 36250636 10.1093/nar/gkac901 PMC9825193

[31] Hang X, Huazhu F, Long Y et al. Unsupervised spatially embedded deep representation of spatial transcriptomics. *Genome Med* 2024;16:12. 10.1186/s13073-024-01283-x 38217035 PMC10790257

[32] Lei L, Han K, Wang Z et al. Attention-guided variational graph autoencoders reveal heterogeneity in spatial transcriptomics. *Brief Bioinform* 2024;25:bbae173. 10.1093/bib/bbae173 PMC11021349 38627939

[33] Ren H, Walker BL, Cang Z et al. Identifying multicellular spatiotemporal organization of cells with spaceflow. *Nat Commun* 2022;13:4076. 10.1038/s41467-022-31739-w PMC9283532 35835774

[34] Zong Y, Tingyang Y, Wang X et al. conST: an interpretable multi-modal contrastive learning framework for spatial transcriptomics. *BioRxiv* 2022;2022–01.

[35] Long Y, Ang KS, Li M et al. Spatially informed clustering, integration, and deconvolution of spatial transcriptomics with GraphST. *Nat Commun* 2023;14:1155. 10.1038/s41467-023-36796-3 36859400 PMC9977836

[36] Wang B, Luo J, Liu Y et al. Spatial-MGCN: a novel multi-view graph convolutional network for identifying spatial domains with attention mechanism. *Brief Bioinform* 2023;24:bbad262. 37466210 10.1093/bib/bbad262

[37] Zhuohan Y, Yifu L, Wang Y et al. ZINB-based graph embedding autoencoder for single-cell RNA-seq interpretations. *Proceedings of the AAAI conference on artificial intelligence* 2022;36:4671–9. 10.1609/aaai.v36i4.20392

[38] David James Fletcher . Estimating overdispersion when fitting a generalized linear model to sparse data. *Biometrika* 2012;99:230–7. 10.1093/biomet/asr083

[39] Maynard KR, Collado-Torres L, Weber LM et al. Transcriptome-scale spatial gene expression in the human dorsolateral prefrontal cortex. *Nat Neurosci* 2021;24:425–36. 10.1038/s41593-020-00787-0 33558695 PMC8095368

[40] Buache E, Etique N, Alpy F et al. Deficiency in trefoil factor 1 (TFF1) increases tumorigenicity of human breast cancer cells and mammary tumor development in TFF1-knockout mice. *Oncogene* 2011;30:3261–73. 10.1038/onc.2011.41 21358676 PMC3141110

[41] Hang X, Huazhu F, Long Y et al. Unsupervised spatially embedded deep representation of spatial transcriptomics. *Genome Med* 2024;16:12. 10.1186/s13073-024-01283-x 38217035 PMC10790257

[42] Wang X, Allen WE, Wright MA et al. Three-dimensional intact-tissue sequencing of single-cell transcriptional states. *Science* 2018;361:eaat5691. 10.1126/science.aat5691 PMC6339868 29930089

[43] Chen A, Liao S, Cheng M et al. Spatiotemporal transcriptomic atlas of mouse organogenesis using DNA nanoball-patterned arrays. *Cell* 2022;185:1777–1792.e21. 10.1016/j.cell.2022.04.003 35512705

[44] Stickels RR, Murray E, Kumar P et al. Highly sensitive spatial transcriptomics at near-cellular resolution with Slide-seqv2. *Nat Biotechnol* 2021;39:313–9. 10.1038/s41587-020-0739-1 33288904 PMC8606189

[45] Sunkin SM, Ng L, Lau C et al. Allen Brain Atlas: an integrated spatio-temporal portal for exploring the central nervous system. *Nucleic Acids Res* 2012;41:D996–D1008. 10.1093/nar/gks1042 23193282 PMC3531093

[46] Alexander Wolf F, Angerer P, Theis FJ. SCANPY: large-scale single-cell gene expression data analysis. *Genome Biol* 2018;19:15. 29409532 10.1186/s13059-017-1382-0 PMC5802054

[47] Shang L, Zhou X. Spatially aware dimension reduction for spatial transcriptomics. *Nat Commun* 2022;13:7203. 10.1038/s41467-022-34879-1 36418351 PMC9684472

[48] Alexander Wolf F, Hamey FK, Plass M et al. PAGA: graph abstraction reconciles clustering with trajectory inference through a topology preserving map of single cells. *Genome Biol* 2019;20:59. 30890159 10.1186/s13059-019-1663-x PMC6425583

[49] Zeng H, Shen EH, Hohmann JG et al. Large-scale cellular-resolution gene profiling in human neocortex reveals species-specific molecular signatures. *Cell* 2012;149:483–96. 10.1016/j.cell.2012.02.052 22500809 PMC3328777

[50] Arnsten AFT, Datta D, Wang M. The genie in the bottle-magnified calcium signaling in dorsolateral prefrontal cortex. *Mol Psychiatry* 2021;26:3684–700. 10.1038/s41380-020-00973-3 33319854 PMC8203737

[51] Souza D M-d, Guest PC, Harris LW et al. Identification of proteomic signatures associated with depression and psychotic depression in post-mortem brains from major depression patients. *Transl Psychiatry* 2012;2:e87–7. 10.1038/tp.2012.13 22832852 PMC3309534

[52] John F, Enwright III, Arion D et al. Differential gene expression in layer 3 pyramidal neurons across 3 regions of the human cortical visual spatial working memory network. *Cereb Cortex* 2022;32:5216–29. 10.1093/cercor/bhac009 35106549 PMC9667185

[53] Arnold M, Morgan E, Rumgay H et al. Current and future burden of breast cancer: global statistics for 2020 and 2040. *The Breast* 2022;66:15–23. 10.1016/j.breast.2022.08.010 36084384 PMC9465273

[54] Benatar T, Yang W, Amemiya Y et al. IGFBP7 reduces breast tumor growth by induction of senescence and apoptosis pathways. *Breast Cancer Res Treat* 2012;133:563–73. 10.1007/s10549-011-1816-4 21997538

[55] Würtz SØ, Würtz SØ, Schrohl A-S et al. TIMP-1 as a tumor marker in breast cancer–an update. *Acta Oncol* 2008;47:580–90. 10.1080/02841860802022976 18465326

[56] Lefranc M-P . Nomenclature of the human immunoglobulin heavy (IGH) genes. *Exp Clin Immunogenet* 2001;18:100–16. 10.1159/000049189 11340299

[57] Leśniak W, Słomnicki ŁP, Filipek A. S100a6–new facts and features. *Biochem Biophys Res Commun* 2009;390:1087–92. 10.1016/j.bbrc.2009.10.150 19891957

[58] Sureshbabu A, Okajima H, Yamanaka D et al. IGFBP5 induces cell adhesion, increases cell survival and inhibits cell migration in MCF-7 human breast cancer cells. *J Cell Sci* 2012;125:1693–705. 10.1242/jcs.092882 22328518

[59] Cleven AHG, Höcker S, Bruijn I B-d et al. Mutation analysis of H3F3A and H3F3B as a diagnostic tool for giant cell tumor of bone and chondroblastoma. *Am J Surg Pathol* 2015;39:1576–83. 10.1097/PAS.0000000000000512 26457357

[60] Morrison K, Challita-Eid PM, Raitano A et al. Development of ASG-15ME, a novel antibody–drug conjugate targeting SLITRK6, a new urothelial cancer biomarker. *Mol Cancer Ther* 2016;15:1301–10. 10.1158/1535-7163.MCT-15-0570 26944921

[61] Mason DY, Cordell JL, Brown MH et al. CD79a: a novel marker for B-cell neoplasms in routinely processed tissue samples. Blood 1995;86:1453–9. 7632952

[62] Horna P, Weybright MJ, Ferrari M et al. Dual T-cell constant $\beta $ chain (TRBC) 1 and TRBC2 staining for the identification of T-cell neoplasms by flow cytometry. *Blood Cancer J* 2024;14:34. 10.1038/s41408-024-01002-0 38424120 PMC10904869

[63] Zhao Y, Huiting S, Shen X et al. The immunological function of CD52 and its targeting in organ transplantation. *Inflamm Res* 2017;66:571–8. 10.1007/s00011-017-1032-8 28283679

[64] Furusato B, Rhim JS. CXCR4 and cancer. *Chemokine receptors*. *Cancer* 2009;31–45. 10.1007/978-1-60327-267-4_219171042

[65] Allgöwer C, Kretz A-L, von Karstedt S et al. Friend or foe: S100 proteins in cancer. *Cancers* 2020;12:2037. 10.3390/cancers12082037 32722137 PMC7465620

[66] May FEB, Westley BR. TFF3 is a valuable predictive biomarker of endocrine response in metastatic breast cancer. *Endocr Relat Cancer* 2015;22:465. 25900183 10.1530/ERC-15-0129 PMC4455223

[67] Caiado H, Conceicao N, Tiago D et al. Evaluation of MGP gene expression in colorectal cancer. *Gene* 2020;723:144120. 10.1016/j.gene.2019.144120 31589964

[68] Yang L, Chen Y, Cui T et al. Desmoplakin acts as a tumor suppressor by inhibition of the Wnt/-catenin signaling pathway in human lung cancer. *Carcinogenesis* 2012;33:1863–70. 10.1093/carcin/bgs226 22791817

[69] De Mezer M, Rogaliński J, Przewoźny S et al. SERPINA3: stimulator or inhibitor of pathological changes. *Biomedicines* 2023;11:156. 10.3390/biomedicines11010156 36672665 PMC9856089

[70] Novak D, Hüser L, Elton JJ et al. Sox2 in development and cancer biology. Semin Cancer Biol 2020;67:74–82. 10.1016/j.semcancer.2019.08.007 31412296

[71] Saadatmand AR, Sramek V, Weber S et al. CaM kinase ii regulates cardiac hemoglobin expression through histone phosphorylation upon sympathetic activation. *Proc Natl Acad Sci* 2019;116:22282–7. 31619570 10.1073/pnas.1816521116 PMC6825262

[72] Orr N, Arnaout R, Gula LJ et al. A mutation in the atrial-specific myosin light chain gene (MYL4) causes familial atrial fibrillation. *Nat Commun* 2016;7:11303. 10.1038/ncomms11303 PMC4832069 27066836

[73] Sauzay C, Petit A, Bourgeois A-M et al. Alpha-foetoprotein (AFP): a multi-purpose marker in hepatocellular carcinoma. *Clin Chim Acta* 2016;463:39–44. 10.1016/j.cca.2016.10.006 27732875

[74] Litingtung Y, Chiang C. Control of Shh activity and signaling in the neural tube. *Dev Dyn* 2000;219:143–54. 10.1002/1097-0177(2000)9999:9999∖(〈∖)::AID-DVDY1050∖(〉∖)3.3.CO;2-H 11002335

[75] Nguyen PD, Hollway GE, Sonntag C et al. Haematopoietic stem cell induction by somite-derived endothelial cells controlled by meox1. *Nature* 2014;512:314–8. 10.1038/nature13678 25119043

[76] Kumar AS, Tian L, Bolondi A et al. Spatiotemporal transcriptomic maps of whole mouse embryos at the onset of organogenesis. *Nat Genet* 2023;55:1176–85. 10.1038/s41588-023-01435-6 37414952 PMC10335937

[77] Kokkinopoulos I, Ishida H, Saba R et al. Single-cell expression profiling reveals a dynamic state of cardiac precursor cells in the early mouse embryo. *PloS One* 2015;10:e0140831. 10.1371/journal.pone.0140831 26469858 PMC4607431

