# Supplementary material for: SpatialDG: a novel spatial domain identification method for spatially resolved transcriptomics data based on dual-graph neural network
Source: Brief Bioinform. 2026 Apr 6;27(2):bbag145. doi: 10.1093/bib/bbag145 (PMC13056729; doi:10.1093/bib/bbag145)
Supplement: Supplement_bbag145 [file supplement_bbag145.pdf]

Supplementary1: Performance comparison of spatial domain identification methods on DLPFC dataset.

Figure S1.

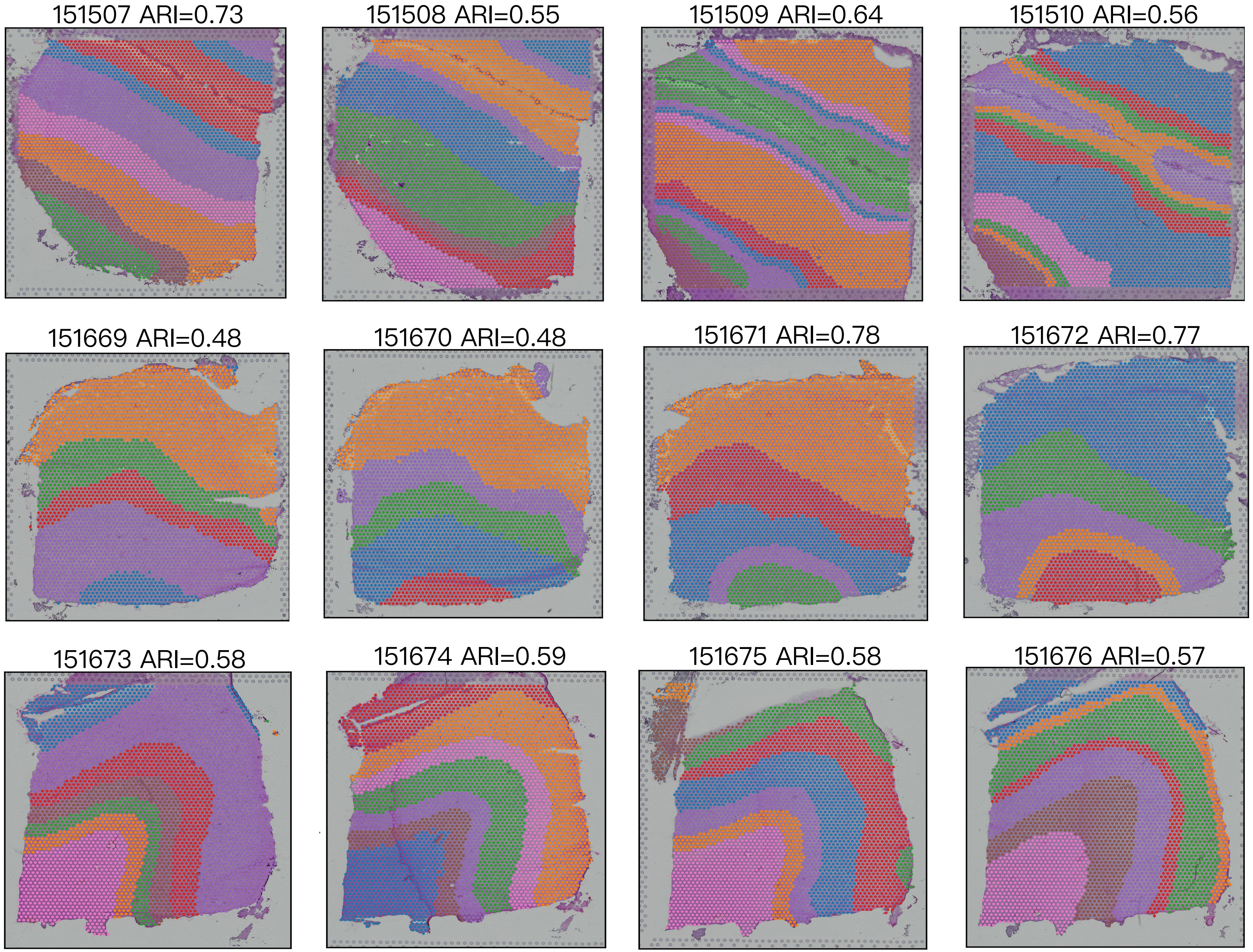

Supplementary2: Spatial domain identification across multiple DLPFC tissue sections.

Table S1.

Slices	SpatialDG	stLearn	GraphST	Spatial-MGCN	STAGATE	DeepST	SEDR
151507	0.74	0.49	0.43	0.63	0.31	0.52	0.27
151508	0.55	0.47	0.47	0.46	0.33	0.51	0.31
151509	0.64	0.44	0.52	0.54	0.43	0.49	0.32
151510	0.56	0.45	0.53	0.51	0.44	0.48	0.35
151669	0.48	0.45	0.57	0.39	0.47	0.47	0.37
151670	0.48	0.32	0.58	0.35	0.49	0.44	0.38
151671	0.78	0.19	0.53	0.60	0.52	0.43	0.41
151672	0.77	0.38	0.55	0.70	0.54	0.42	0.43
151673	0.58	0.35	0.61	0.61	0.57	0.41	0.46
151674	0.59	0.37	0.57	0.60	0.58	0.44	0.47
151675	0.58	0.36	0.67	0.54	0.60	0.52	0.49
151676	0.57	0.38	0.54	0.58	0.62	0.46	0.57

Supplementary3: Stability analysis of SpatialDG across multiple random seeds.

Figure S2.

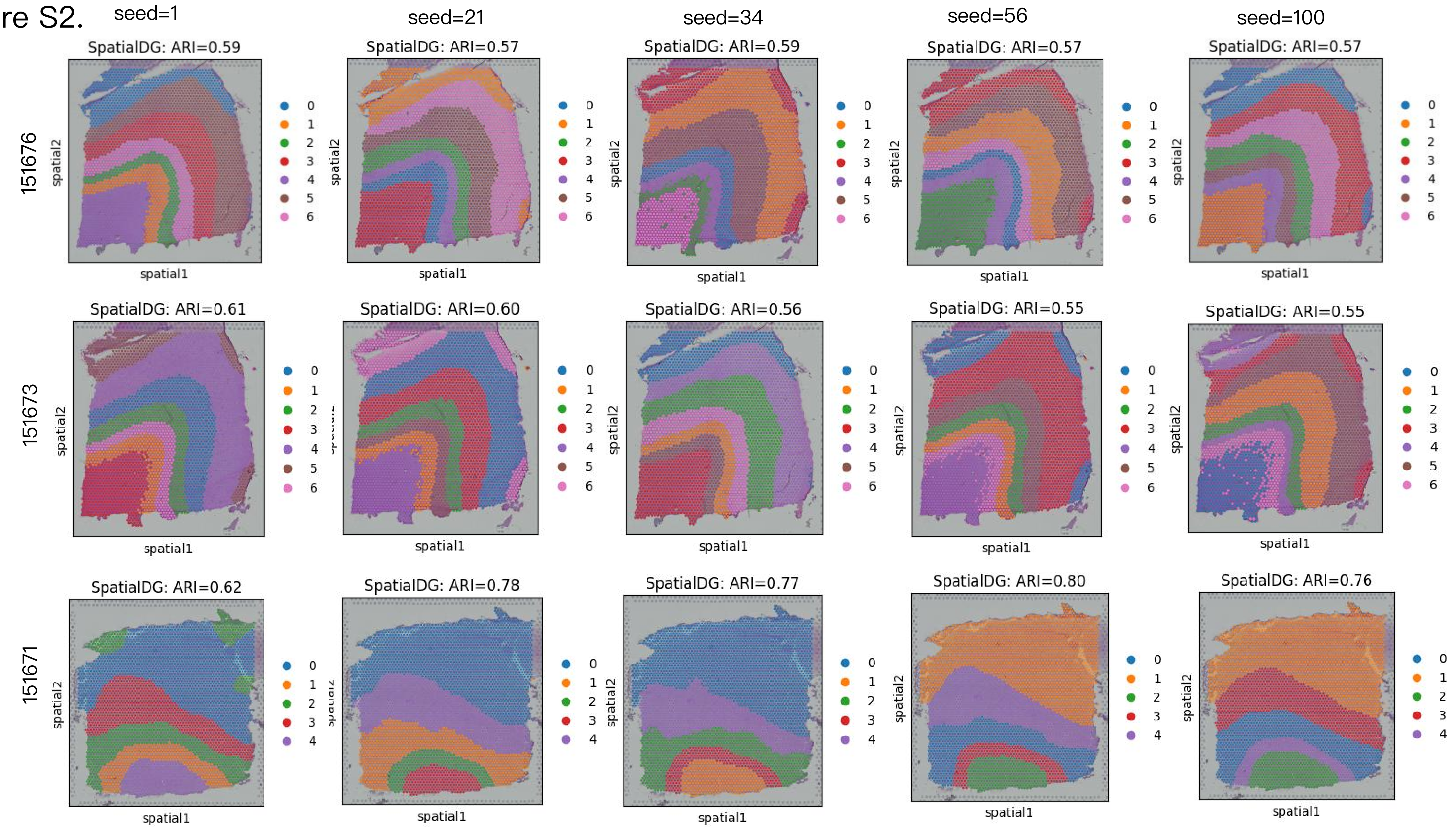

Supplementary3: Stability analysis of SpatialDG across multiple random seeds.

Table S2.

Slice ID	Seed 1	Seed 21	Seed 34	Seed 56	Seed 100	Mean ± SD
151676	0.59	0.57	0.59	0.57	0.57	0.578 ± 0.011
151673	0.61	0.60	0.56	0.55	0.55	0.574 ± 0.029
151671	0.62	0.78	0.77	0.80	0.76	0.746 ± 0.072

The model was trained with 5 independent random seeds (1, 21, 34, 56, 100) on three representative DLPFC slices.
